# Alterations in Tau Metabolism in ALS and ALS-FTSD

**DOI:** 10.3389/fneur.2020.598907

**Published:** 2020-11-23

**Authors:** Michael J. Strong, Neil S. Donison, Kathryn Volkening

**Affiliations:** ^1^Molecular Medicine, Schulich School of Medicine and Dentistry, Robarts Research Institute, Western University, London, ON, Canada; ^2^Department of Clinical Neurological Sciences, Schulich School of Medicine and Dentistry, Western University, London, ON, Canada; ^3^Neuroscience Graduate Program, Western University, London, ON, Canada

**Keywords:** TDP-43, phosphorylation, traumatic encephalopathy, motor neuron, frontotemporal

## Abstract

There is increasing acceptance that amyotrophic lateral sclerosis (ALS), classically considered a neurodegenerative disease affecting almost exclusively motor neurons, is syndromic with both clinical and biological heterogeneity. This is most evident in its association with a broad range of neuropsychological, behavioral, speech and language deficits [collectively termed ALS frontotemporal spectrum disorder (ALS-FTSD)]. Although the most consistent pathology of ALS and ALS-FTSD is a disturbance in TAR DNA binding protein 43 kDa (TDP-43) metabolism, alterations in microtubule-associated tau protein (tau) metabolism can also be observed in ALS-FTSD, most prominently as pathological phosphorylation at Thr^175^ (pThr^175^tau). pThr^175^ has been shown to promote exposure of the phosphatase activating domain (PAD) in the tau N-terminus with the consequent activation of GSK3β mediated phosphorylation at Thr^231^ (pThr^231^tau) leading to pathological oligomer formation. This pathological cascade of tau phosphorylation has been observed in chronic traumatic encephalopathy with ALS (CTE-ALS) and in both *in vivo* and *in vitro* experimental paradigms, suggesting that it is of critical relevance to the pathobiology of ALS-FTSD. It is also evident that the co-existence of alterations in the metabolism of TDP-43 and tau acts synergistically in a rodent model to exacerbate the pathology of either.

## Introduction

Our understanding of the pathobiology of amyotrophic lateral sclerosis (ALS) has evolved dramatically since its first description as a clinicopathological entity in 1874 by Charcot as a disorder of progressive muscular atrophy associated with spasticity (1). ALS was once considered a disorder restricted to the degeneration of the descending supraspinal motor neurons and those lower motor neurons subserving bulbar and spinal motor functions, with specific sparing of a number of functions (including cognition). This concept, however, has been replaced by the understanding that ALS is a multisystems disorder for which motor neuron dysfunction is one aspect of a much larger picture (2, 3).

Given this, it is not surprising that ALS is now considered to be syndromic with clinical manifestations that reflect not only a range of underlying genetic and biochemical impairments consistent with prominent disruptions in RNA metabolism and protein degradation pathways, but also with considerable non-motor involvement (4). This latter aspect has become increasingly evident as our recognition and understanding of the neuropsychological manifestations of ALS has grown. While there has also been remarkable growth in our understanding of the perturbed biochemistry underlying this aspect of the disorder, there remains a tremendous amount that is unknown. In this review, we will discuss the nature of the phenotypic heterogeneity of ALS as it applies to associated syndromes of frontotemporal dysfunction that can be associated with ALS, explore the nature of the proteinopathies that are thought to underlie the process including a critical analysis of perturbations in microtubule associated protein tau (tau) metabolism, and consider a conceptual framework in which synergism amongst co-expressed toxic proteins can drive disease phenotype.

## Contemporary Conceptualization of the Clinical and Neuropathological Features of ALS

The classic clinical and neuropathological description of ALS hinges on the evidence of motor system degeneration in which the loss of lower motor neurons drives progressive muscle atrophy while the loss of upper motor neurons is predominantly, but not exclusively, manifested as spasticity. The loss of motor function culminates in respiratory failure in the majority of individuals within 3 and 5 years of symptom onset, although there is wide variability in survivorship (5). Degenerating motor neurons in ALS classically bear the neuropathological hallmark of neuronal cytoplasmic inclusions (NCIs) composed largely of cytoskeletal proteins, predominantly of the neuronal intermediate filament family (6). Ubiquitination of these proteins is consistent with the concept of impaired proteasomal degradation in ALS as a key pathological process.

This classical conceptualization of ALS has been significantly altered through two fundamental observations: firstly, that widespread frontotemporal dysfunction can occur in a significant proportion of patients (7–11); and secondly, that a fundamental alteration in RNA metabolism, at multiple levels, is a core biological process for the majority of ALS cases (12). This latter postulate is supported by the observation of NCIs composed of a broad range of RNA binding proteins not only within degenerating motor neurons, but within cortical and subcortical neurons in ALS. It was these observations that have now provided a unifying linkage between the two major clinical aspects of ALS–progressive motor neuron degeneration and frontotemporal dysfunction. The key breakthrough that led to this transformative understanding of ALS was the discovery that TDP-43 (TAR DNA binding protein, or transactive response DNA binding protein 43 kDa) accumulates in degenerating motor neurons and within degenerating cortical neurons in frontotemporal lobar degeneration (FTLD) (13, 14). This protein is a DNA/RNA binding protein that is vital to stress and injury responses and is intricately involved in the regulation of RNA metabolism (15, 16). While pathological neuronal and glial cytoplasmic and nuclear inclusions of TDP-43 are now considered to be the neuropathological hallmark of ALS, multiple RNA binding proteins can aggregate as pathological NCIs in ALS, often within the same motor neurons, and often colocalizing to the same aggregates (17–24). This lends support to the broader concept of ALS as a disorder of RNA metabolism in which a diverse array of RNA binding proteins can be involved, likely in several different mechanisms ranging from mutations resulting in gain- or loss-of-function of RNA binding proteins [including fused in sarcoma (FUS) and TDP-43] to also including those proteins in which ALS-associated mutations confer novel RNA interacting capacity [for instance as observed with mutations in copper/zinc superoxide dismutase (mtSOD1)] (25–27). This is consistent with the conceptualization that ALS is syndromic and reflective of a significant biological heterogeneity underlying not just its pathobiology, but also its phenotypic expression.

Nowhere is this more evident than in our understanding and acceptance that individuals with otherwise classical ALS can be affected by a range of neuropsychological, behavioral, speech, and language deficits. These can range from mild to severe impairment, including frontotemporal dementia (FTD) (28, 29). These deficits are encapsulated in the most recent diagnostic criteria under the rubric of the “frontotemporal spectrum disorder” of ALS (ALS-FTSD) (7). Applying these criteria, less than half of ALS patients have a pure motor neuron disorder in which there is no evidence of degeneration outside of the motor system, and for whom the El Escorial or Awaji criteria are fully met (30, 31). Amongst the remaining patients, ~15% will also suffer from, or will have presented with, a FTD that meets the Rascovsky et al. criteria for diagnosis, including progression (32, 33). The remaining patients will exhibit one or more features of behavioral impairment, cognitive or executive dysfunction, speech, and/or language impairments, and in some cases, a mixture thereof but at levels insufficient for a diagnosis of FTD. Approximately 2% of ALS patients can also be affected by a non-FTD dementia such as Alzheimer's disease or vascular cognitive impairment. Finally, there is also a subgroup of patients with frontotemporal lobar degeneration (FTLD, the neuropathological correlate of the FTD) in whom motor neuron degeneration, typically marked by pathological TDP-43 inclusions, is only obvious postmortem.

It is this broad range of clinical deficits that has led to the current thinking that ALS and FTD are but two points on a disease continuum, with ALS-FTSD residing between these two extremes (34). Where along this continuum a patient will manifest is simply the clinical reflection of where the greatest burden of disease is impacting at that moment in time, both in terms of the extent of pathology present but also on the nature of disruption of the affected neural networks. This conceptualization is supported by the observation that the neuropathological hallmark of the vast majority of ALS cases, regardless of the presence or absence of ALS-FTSD, is the presence of pathological cytoplasmic TDP-43 inclusions reflective of disruption in the metabolism of TDP-43 and thus sharing a feature in common with a subset of FTD patients (35, 36).

To this end, the pathological characterization of FTLD is based on the primary protein associated with the neuropathology. Using this classification, approximately half of which are FTLD-TDP (37) and slightly less than half are tau predominant pathology (FTLD-tau) (36). The remaining minority of cases demonstrate neither, but rather pathology driven by alterations in the FET family of proteins [FUS; Ewing's Sarcoma (EWS) and TATA-binding protein-associated factor 15 (TAF15)] metabolism or with evidence for impaired ubiquitin-conjugated of proteins (FTLD-UPS) (37). The challenge with this categorization is that there is increasing evidence to support overlaps amongst these pathologies, and in particular between TDP-43 and tau (38). As will be discussed, there is evidence to suggest that this underlies the pathobiology of ALS-FTSD.

## Pathological Tau Metabolism in ALS-FTSD

**Figure 1:**
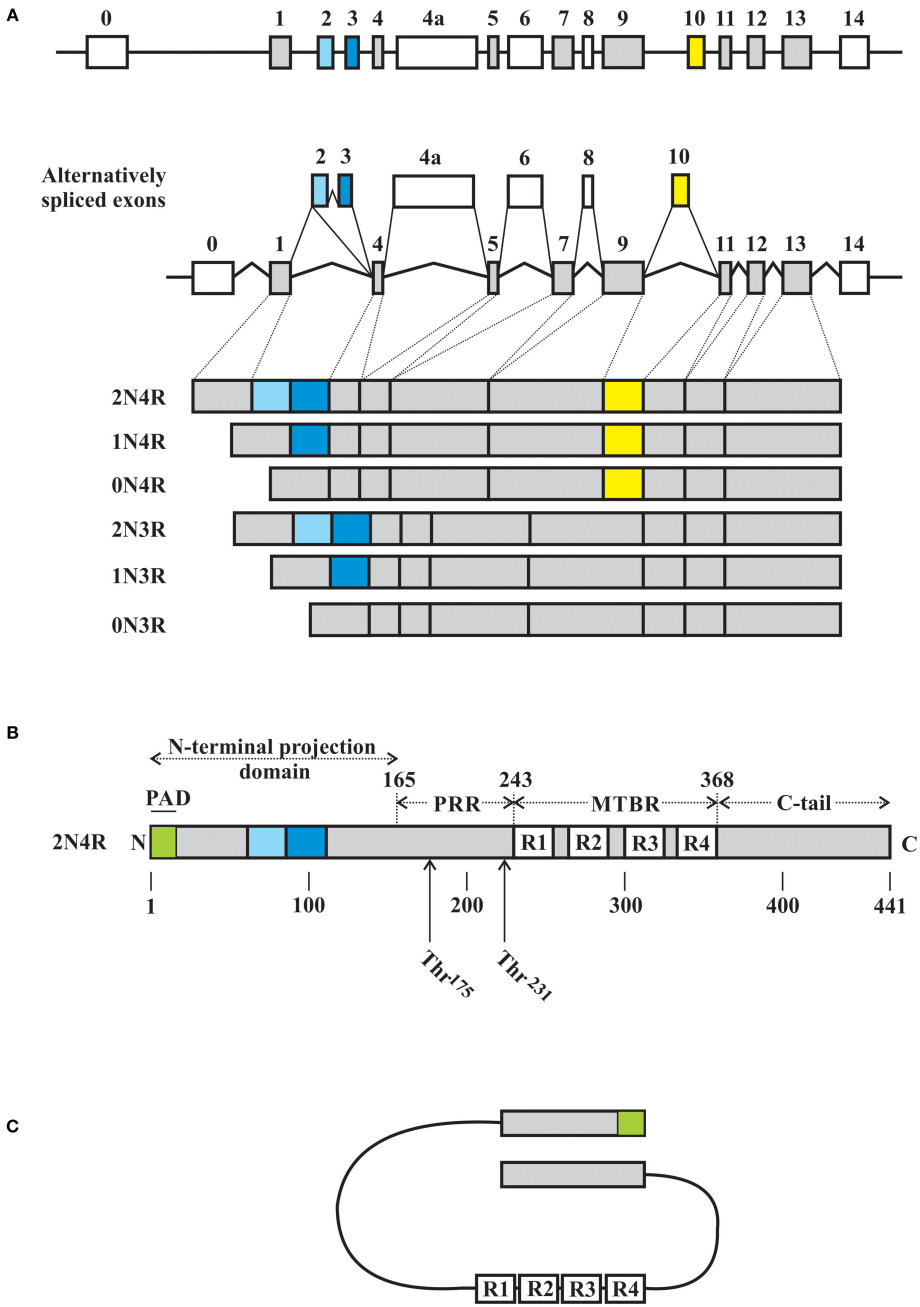
Schematic representations of *MAPT* gene and tau. **(A)** Tau is encoded by a single gene on chromosome 17q.31, with 16 exons, including the alternatively spliced exons 2, 3, 4a, 6, 8, and 10. Start and stop codons are located in exons 1 and 13, respectively. Exons 2, 3, and 10 are alternatively expressed in the adult human brain, giving rise to 6 tau isoforms based on the inclusion of 0, 1, or 2 N-terminus inserts encoded by exons 2 or 3 (exon 2 can be alternatively spliced independent of exon 3, while exon 3 is always expressed with an exon 2 encoded insert) with 3 or 4 microtubule binding repeats (MTBR) depending on the expression of an exon 10 encoded repeat. As such, the 6 isoforms are described as 0N, 1N, or 2N with either 3R or 4R MTBRs. Exon 4A and 6 are expressed in the peripheral nervous system giving rise to additional tau isoforms. To date, exon 8 does not appear to be expressed. **(B)** Using numbering based on the 2N4R isoform of tau of 441 amino acid residues, tau has four major domains, including the N-terminus domain (residues 1–165) with the phosphatase activating domain (PAD) localized to the extreme N-terminus region, a proline rich domain (PRR, residues 166–242), a microtubule assembly domain (residues 243–367) consisting of either 3 or 4 microtubule binding repeats (R1 thru R4, each of ~31 amino acid residues), and a C-terminus domain (residues 368–441). **(C)** Schematic representation of the proposed paperclip conformation of soluble tau in which the N-terminus domain, C-terminus domain, and MTBR are in close approximation. Schematic illustrations based on the following references: (43–47).

Tau is an intrinsically disordered, highly conserved protein that is primarily enriched in axons of central neurons and whose primary function is to stabilize and promote microtubule stability (39–42). The *MAPT* gene located on chromosome 17q21 that encodes for human tau contains 16 exons, of which exons 2 and 3 encode for N-terminus insertions of 29 amino acids in length while exon 10 encodes for one of four microtubule binding repeats (Figure 1). Through alternative splicing of the tau mRNA, tau can therefore exist as 6 isoforms ranging from 352 to 441 amino acid residues with apparent molecular weights of 48–67 kDa (predicted 36–45 kDa without modifications) and characterized by the presence or absence of one or two N-terminus inserts (yielding 0N, 1N, or 2N isoforms) accompanied by the expression of either 3 or 4 microtubule binding repeats (3R or 4R) (48). Tau is a largely unstructured soluble protein that *in vitro* takes on a “paperclip” conformation such that the N-terminus, C-terminus, and microtubule binding region (MTBR) are in close approximation (Figure 1C) (43–46, 49, 50). The relative expression of 3R to 4R isoforms is highly regulated and when altered is associated with a range of neurodegenerative disease states characterized by FTLD with pathological tau deposition (36). Tau undergoes extensive post-translational modification, with phosphorylation arguably being the most crucial to its interaction with microtubules (48, 51). Site specific tau phosphorylation modulates the interaction between tau and microtubules, with pathological phosphorylation dissociating the two and giving rise to elevated soluble tau isoforms which are then free to dimerize into stable tau oligomers, which then continue to polymerize into protomers and lead to fibril formation.

While the primary function of tau is to promote microtubule stability, tau also appears to be involved in several other non-cytoskeletal related activities within cells. Phosphorylation of tau plays a role in determining the localization of tau throughout neurons, with soma localized tau exhibiting high levels of phosphorylation, and a gradual loss of phosphorylation occurring as tau is located closer to the growth cone of an axon (52). It was originally thought that tau phosphorylation was altered as tau was transported through the cell; however, evidence also supports a model in which new tau is synthesized and immediately phosphorylated, with this pattern of phosphorylation dictating where in the cell that tau will ultimately be localized (49, 53, 54).

A tau isoform that lacks phosphorylation at S^195^-S^202^ is localized to the nucleus, where it is involved in DNA binding (either through association with AT-rich DNA, or with the minor groove of AT-rich DNA sequences) (55, 56), heterochromatin stability, transcription repression of ribosomal DNA, long non-coding RNA production, or possibly in repressing gene expression (55, 57, 58). The isoform of tau expressed is also important in that compartmentalization appears to prefer one isoform type to another, with isoforms bearing 0N or 2N preferentially located outside of the nucleus, while 1N isoforms are found preferentially in the nucleus (59).

In response to cell stress, nuclear tau translocates to the nucleolus, where it is postulated to increase RNA binding protein interactions with TIA1 (T-cell intracellular antigen) (60). Cell stress also elicits the formation of stress granules (SG) within neurons. TIA1 translocates to SG in the cytosol, where these TIA1 positive SG have been detected to localize with tau that is highly phosphorylated, or “hyper” phosphorylated. However, not all SG are TIA1 positive, and other populations of SG do not appear to colocalize with hyperphosphorylated tau, for example the G3BP1-positive SG (61). SG formation has been noted to be upregulated in ALS, with both TDP-43 and FUS proteins playing important roles in the formation and maintenance of SG [reviewed in (62)]. Proteins with low complexity/highly disordered domains appear to be integral to SG formation, and dysregulation of the expression of these proteins, the presence of mutations, or alterations in the localization of these proteins appear to have a profound effects on SG dynamics in neurodegenerative disease including ALS and FTLD [reviewed in (63, 64)]. The role(s) of tau in SG formation is unclear; however evidence shows that if tau is overexpressed there is an increase in SG formation (65–67). There is also evidence suggesting that the deposition of tau can induce the condensation of RNA binding proteins through liquid-liquid phase separation (LLPS). LLPS has been shown to involve highly disordered protein domains [including the DNA/RNA binding protein TDP-43 (68)] that tend to fall into an insoluble state spontaneously when in high enough concentrations. Tau can experience LLPS and as such may induce the formation of toxic tau conformations (69–72). It would not be unreasonable to postulate that tau may help to initiate LLPS of RNA binding proteins that are contained within the same SG. Consistent with this concept, there is evidence that the RNA binding proteins Musashi 1 and 2 (MSI 1 and MSI 2, respectively), both highly expressed in mature neurons, form intranuclear complexes with oligomeric tau proteins in a concentration dependant manner (73). In doing so, these complexes significantly impair nucleocytoplasmic transport of NLS-containing cargo, a process that has been postulated to fundamental to the pathobiology of ALS and FTD (74). It is of direct relevance therefore that both intracellular and extracellular MSI 1 and MSI 2 complexes are present in both ALS and FTD cortical tissue, and that this occurs in the presence of increased oligomeric tau (73). It is often inferred that the metabolism of tau protein is altered in any given disease process based largely on the presence of tau immunoreactive NCIs; the finding that this immunoreactivity is also for specific tau phospho epitopes normally observed in the presence of a disease state supports this. This is further augmented by the biochemical characterization of the tau protein following separation into detergent soluble or insoluble fractions, with characterization then based on the relative preponderance of either or both the 3R or 4R isoforms. However, the deposition of tau protein can be either a primary process as in neurodegeneration or a secondary process as in a stress response (60, 61, 75).

In the following, we will examine the neuropathological, biochemical, and experimental evidence in support of pathological tau metabolism amongst a subset of ALS patients.

### Neuropathological and Biochemical Evidence of Altered Tau Metabolism in ALS

Tau dysmetabolism has been most clearly documented in the Western Pacific variant of ALS in which affected individuals developed either a Parkinson-Dementia-ALS complex or ALS alone, at rates several thousand fold greater than observed amongst the rest of the world (76). The characteristic neuropathological feature was the widespread deposition of tau protein throughout the neuroaxis, including spinal motor neurons (77, 78). This latter feature is not traditionally observed in other ALS variants with the exception of those individuals with chronic traumatic encephalopathy (CTE) who also develop clinical and electrophysiological features consistent with a diagnosis of ALS (CTE-ALS) (79–81).

Beyond this, the evidence for alterations in tau metabolism in ALS has been scant and largely limited to individual case reports or unique inherited disorders in which ALS has been described amongst a broader range of neurological and neuropathological deficits or in association with rare *MAPT* mutations (82–87). However, two instructive exceptions drawn from the spectrum of genetic mutations associated with ALS, ALS-FTD and FTD are the pathological hexanucleotide expansions of C9*orf* 72 and the ALS-associated mutations of FUS (88–90). Both provide further support for the concept of a biological continuum underlying the pathogenesis of ALS and FTD in addition to insights into mechanisms of altered tau metabolism.

Pathological hexanucleotide repeat (GGGGCC)_n_ expansion of C9*orf* 72 is the most common hereditary molecular cause of ALS, ALS-FTD and FTD. The neuropathological hallmark of the associated FTLD includes foci of dinucleotide-peptide repeats (DPRs) in specific neuronal subpopulations in association with more widespread pathological TDP-43 deposition [reviewed in (36, 91)] in the absence of an associated tauopathy or when present, as a non-specific finding (92). Although the exact mechanism by which these pathological expansions give rise to a toxic variant of C9*orf* 72, there is evidence to support a role in fundamentally altering RNA metabolism through a variety of avenues [reviewed in (91)]. However, there is also emerging evidence that pathological C9*orf* 72 expansion may act in a synergistic manner to increase the propensity for tau pathology by increasing cdk5-mediated tau phosphorylation, including pathological phosphorylation (93–95).

In contrast, mutations in FUS are an uncommon molecular cause of familial ALS, rarely observed in sporadic ALS, and found in only a single case report in consanguineous twins with FTD (17, 18, 90, 96–98). While FUS has an array of both cytoplasmic and nuclear functions, it plays a critical role in regulating alternative splicing through its association with the spliceosome, including in regulating the physiological splicing of *MAPT* exon 10 (99). The loss of FUS activity results in the preferential inclusion of exon 10 and an increased expression of 4R tau (100). FUS forms a complex with splicing factor, proline- and glutamine-rich (SFPQ) through an RNA-dependant interaction such that the loss of either FUS or SFPQ results in the preferential expression of 4R tau and an accompanying increase in ptau expression, including pathological tau isoforms (101–103). The importance of this is highlighted by the finding of reduced interactions between FUS and SFPQ across a broad range of FTLDs, including ALS-FTD (102). The recent report of a single atypical case of sporadic ALS manifesting with predominantly upper motor neuron dysfunction with extrapyramidal features who at autopsy demonstrated pyramidal neuron FUS basophilic inclusions that colocalized with AT8 tau immunoreactivity in motor neurons, in addition to a 4R predominant tauopathy, provides support for such a proposed role of FUS in contributing to alterations in tau metabolism in ALS (104).

More recently, a 17 kDa neurotoxic tau fragment (tau^45−230^) has been observed in extracts of both the brain and ventral spinal cord of sporadic ALS patients, but not in controls (105, 106). This fragment is generated by calpain cleavage of full length tau and has been observed both *in vitro* and *in vivo* across a broad range of tauopathies (107, 108).

Both tau immunoreactive inclusions and pathological tau phosphorylation disproportionate for age have been observed in ALS patients affected with cognitive or executive dysfunction (ALSci), typically in the form of dystrophic neurites, neurofibrillary tangle-like structures and pre-tangles, neuritic granules, and tau-immunoreactive tufted astrocytes (109–111). Tau isolated from mesial frontal cortex and subcortical at postmortem demonstrates a significantly greater propensity for fibril formation in a thioflavin S assay, partitions into the sarkosyl-insoluble fraction as all 6 isoforms (distinguishing it from Alzheimer's disease tau, 3R or 4R tau disorders), and is pathologically phosphorylated at Thr^175^ (pThr^175^tau) (112). Using a polyclonal antibody recognizing pThr^175^tau, widespread tau deposition, sparing the motor neurons, was observed to be in association with a diffuse increase in TDP-43 immunoreactivity (110). In the same study, it was also observed that of those patients without evidence of neuropsychological impairment, approximately half demonstrated pThr^175^tau immunoreactive pathology although significantly less pronounced than observed in those with evidence of ALS-FTSD. Of note, the active isoform of GSK3β (pGSK3β) was also upregulated in those regions most extensively affected with tau pathology (113). The observation of pThr^175^tau pathology was subsequently confirmed in a separate cohort of motor neuron disease (MND) patients with concomitant FTD, including approximately 10% with non-descript neuronal aggregates and as with the earlier study, associated with pathological TDP-43 expression (114). Although not examined for the pThr^175^tau phospho epitope, ptau (pSer^396^, pSer^214^, and pSer^404^) has been described in ALS cervical spinal cord and motor cortex in the cytosol and nuclei of motor neurons as diffuse immunoreactivity in the absence of fibril formation (115). The presence of nuclear tau in this latter series may be reflective of a neuronal stress response although no comment was made with respect to the presence or absence of nucleolar tau staining. To date, no other tau phospho-epitopes have been examined or characterized in either ALS or ALS-FTSD to this extent.

In addition to this evidence supporting alterations in tau metabolism in sporadic ALS, a subset of CTE patients (in whom a tauopathy is the neuropathological hallmark) will have evidence of ALS either antemortem with clinical or electrophysiological evidence of diffuse motor neuron degeneration, or at autopsy (80, 81, 116). Amongst these patients, pThr^175^tau, pThr^231^tau, and oligomeric tau (T22) immunoreactive inclusions are readily observed in both cortical and spinal motor neurons (79).

Given the broad array of pathological site specific tau phospho epitopes associated with a range of neurodegenerative disorders, the observation of pThr^175^tau immunoreactive inclusions suggests but does not prove that phosphorylation at this epitope is also pathologically important amongst a number of tauopathies (117). However, pThr^175^tau is not observed in fetal human tissue although tau exhibits significantly higher molar phosphorylation in the fetus, nor is it observed in the healthy aged (117, 118). Hence, its presence suggests a role in the genesis of pathological tau fibrils.

Elevations in cerebrospinal fluid tau and/or TDP-43 have been widely documented and suggested as potential biomarkers for ALS although for the former these have in general reflected total tau (119, 120). However, others have failed to find similar increases in either CSF total tau or ptau levels in ALS patients (121–124). Thus, it remains unclear if CSF tau can be used as a surrogate marker for a concomitant tauopathy in ALS.

### Experimental Evidence in Support of Alterations of Tau Metabolism in ALS

**Figure 2:**
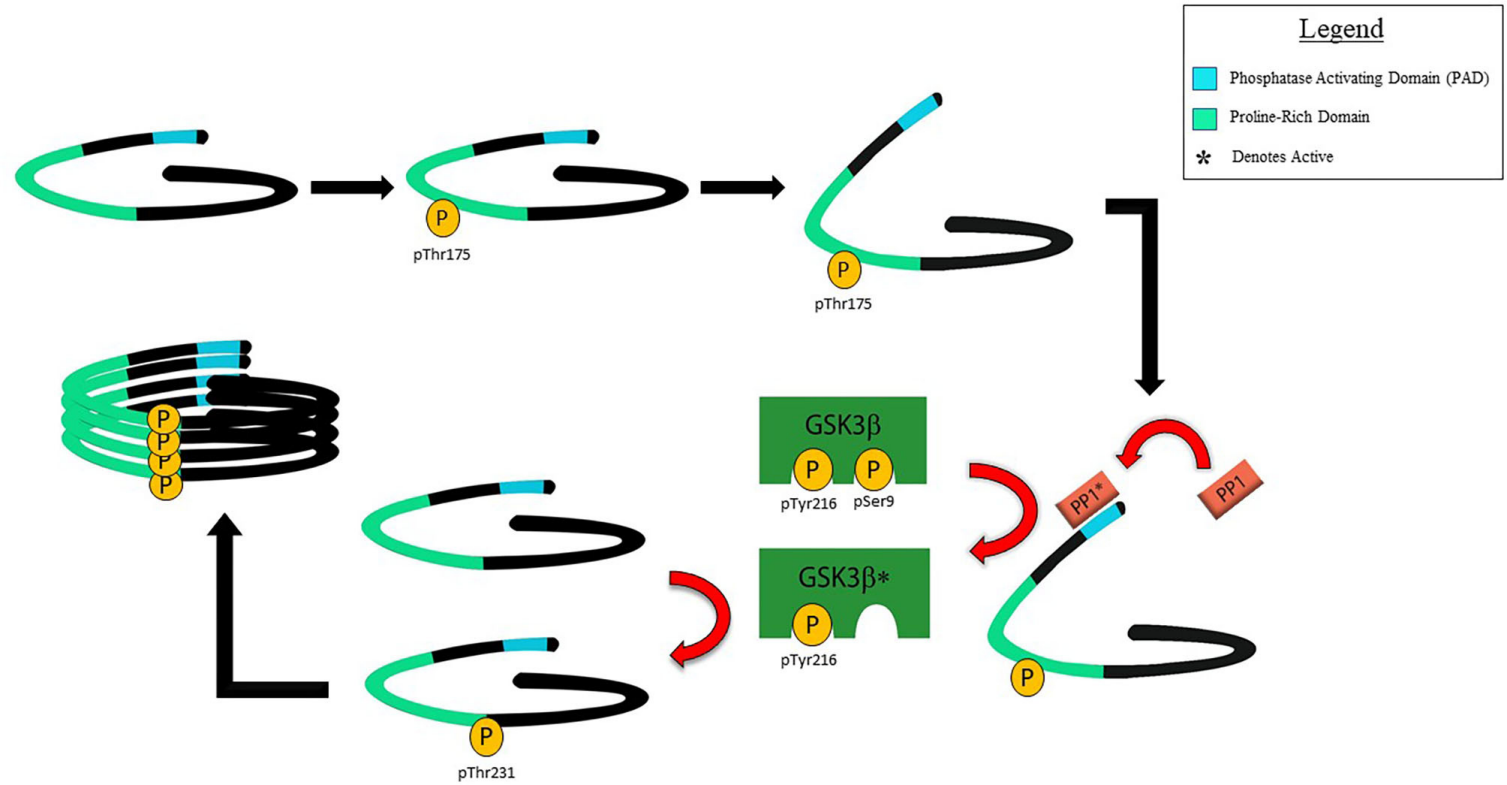
Schematic of proposed mechanism for the toxicity of pThr^175^tau. Under normal physiological conditions, tau is thought to exist in a closed paperclip conformation. Following phosphorylation of Thr^175^ by a kinase or kinases yet to be defined, the closed paperclip structure opens sufficiently to allow for exposure of the PAD which in turn activates protein phosphatase 1 (PP1). PP1 in turn dephosphorylates Ser^9^ of GSK3β, allowing for increased pGSK3β by unmasking pTyr^216^. pGSK3β in turn phosphorylates Thr^231^tau in a process that does not require priming by phosphorylation of Ser^235^tau. It is not known whether pThr^231^ exists in either the *cis* or *trans* isoform, but our evidence suggests that the presence of pThr^231^ is associated with increased tau oligomer formation and its subsequent polymerization into tau fibrils.

Supporting the hypothesis of pathological significance of pThr^175^tau, the introduction of a pseudophosphorylated 2N4R human tau construct mimicking pThr^175^tau (Thr^175^Asp-tau) induces pathological tau fibril formation in both HEK293T and Neuro2a cells, significantly more so than when either wild-type human tau or tau in which phosphorylation at Thr^175^ was inhibited (Thr^175^Ala-tau) were expressed (125). The induction of pathological tau fibril formation *in vitro* was dependent on up-regulation of pGSK3β activity, which in turn induced phosphorylation at Thr^231^tau (pThr^231^tau)—one of several key phosphorylation sites in the cascade of driving tau oligomer formation (Figure 2) (126). The activation of GSK3β is dependent on the dephosphorylation of pSer^9^ which removes inhibition, allowing for GSK3β activity on its substrate (127, 128). We have shown that the phosphorylation of Thr^175^ of tau is associated with an enhanced exposure of the N-terminus of tau, increasing access to the tau phosphatase activating domain (PAD) located at amino acids 2–18 (129, 130). PAD exposure activates protein phosphatase-1 (PP1) which dephosphorylates pSer^9^ of GSK3β enhancing GSK3β activity through a relative increase in exposure of pTyr^216^ while phosphorylation of tau residue 18 (Y18) by the non-receptor tyrosine kinase fyn inhibits the PP1-GSK3β cascade (129, 131). While it remains unknown how tau phosphorylation at Thr^175^ leads to an increased PAD exposure, Thr^175^ resides within the proline-rich domain of tau in which the phosphorylation of other amino acids induces an opening of the tau paperclip conformation, leading to increased PAD exposure (43, 46, 47, 132). It is reasonable to anticipate that pThr^175^ would act similarly.

PAD exposure has been observed to be a common feature of the pathological inclusions across a broad range of tauopathies (133) and has been associated with neurotoxicity through the inhibition of fast axonal transport as an early contribution to the process of neurodegeneration (130, 133–135). In this mechanism, PAD exposure and activation of GSK3β results in phosphorylation of the kinesin light chain which promotes the release of cargo from kinesin 1 (130).

In addition, pThr^231^tau can exist in either *trans* or *cis* conformation (*trans*-pThr^231^ and *cis*-pThr^231^, respectively), in which *cis*-pThr^231^ is specific to the induction of tau pathology and cannot be readily degraded, in contrast to *trans*-pThr^231^ (136). Under normal physiological conditions, the isomerase peptidyl-propyl *cis*-*trans* isomerase NIMA-interacting 1 (PIN1) catalyzes the isomerization of *cis*-pThr^231^ to *trans*-pThr^231^, allowing for the dephosphorylation of this site by PP2A (protein phosphatase 2) and the subsequent ability for tau to exert its physiological activity in microtubule polymerization (137, 138). The pathological relevance of *cis*-pThr^231^ is highlighted by its observation in association with pathological tau deposition in AD and CTE (both experimental and human) (139). In accordance to this, it has also been demonstrated that there are reduced levels of PIN1 in human AD brains, resulting in decreased activity and thus the inability to isomerize *cis*-tau (138, 140). Additionally, genetic polymorphisms resulting in a decrease in PIN1 levels are associated with an increased risk of late-onset AD, whereas another polymorphism which leads to increased PIN1 expression has a neuroprotective effect (141, 142). The relative expression of *cis* vs. *trans*-tau and PIN1 activity in ALS or ALS-FTSD has not been examined to date.

### *In vivo* Evidence of pThr^175^ Toxicity

It had been previously observed that the use of somatic gene transfer of tau harboring the P301L mutation that is causal for frontotemporal dementia with parkinsonism (FTDP-17) using a recombinant adeno-associated virus (rAAV9) injected into the hippocampus of rats induced both tau pathology and impairments in spatial working memory (143). Given this, we examined whether Thr^175^Asp-tau, when inoculated using somatic gene transfer with rAAV9 could similarly induce both pathology and behavioral deficits in rats. We observed that when Thr^175^-Asp tau was inoculated into the hippocampus of young adult female Sprague-Dawley rats, tau pathology was evident as early as 1 month post inoculation and consistently so by 1 year post-inoculation (144). While this was not associated with a behavioral or motor phenotype, it was observed that the burden of neuropathology was localized to the CA2 region, a region for which the testing paradigm employed was insensitive. It was of note however that the induction of tau pathology was accompanied by an upregulation of TDP-43 expression in this model.

Given the observation of pThr^175^tau, pGSK3β, pThr^231^tau, and oligomeric tau in affected neurons of individuals with CTE-ALS, we also examined whether a single subconcussive injury administered to Sprague-Dawley rats would also induce pathological tau fibril formation. Indeed this was the case, suggesting that this specific pathway of pathological tau fibril formation was a primary event (79).

As has been alluded to, concomitant pathologies of both TDP-43 and tau are increasingly recognized, including AD and the aforementioned ALS, CTE-ALS and *in vivo* models of pThr^175^ tau pathology (145–147). To begin to understand how these two pathologies inter-relate, we repeated the somatic gene transfer AAV9 hippocampal inoculums in rats also expressing the ALS-associated mutant human TDP-43 (TDP-43^M337V^). In these rats, constitutive cholinergic neuronal expression was driven by a ChAT promoter with a tTA-dependant tetracycline response element driver (148, 149). In the presence of doxycycline, no mtTDP-43 expression occurred whereas ChAT-tTA/TRE-TDP-43^M337V^ rats, in the absence of doxycycline, developed a fulminant motor phenotype with prominent motor neuron NCI formation. Six months following stereotactic hippocampal inoculums of rAAV9 GFP-tagged tau^T175D^2N4R, a 50% withdrawal of doxycycline was undertaken. Rats were sacrificed 30 days later. Within 3 weeks, all ChAT-tTA/TRE-TDP-43^M337V^ rats had developed motor deficits. At 30 days, a 2-fold increase in the extent of tau pathology was observed in the hippocampus of those rats inoculated with the pseudophosphorylated tau. This was associated with immunoreactivity to a monoclonal antibody recognizing tau phosphorylated at Ser^202^ and Thr^205^ (AT8) (150), suggesting that the tau construct had been further modified to a pathological tau phospho-isoform in the presence of the mutant TDP-43. There was also a trend toward an increase in the extent of spinal motor neuron pathology, a finding that may serve to explain the shortened survival in ALS-FTSD patients when contrasted to patients with ALS alone (151).

This inter-relationship between tau and TDP-43 has been further characterized in a murine model of CTE using a repetitive mild traumatic brain injury (rmTBI) in C57Bl6 mice using a repeated weight drop. In this model, a prominent tauopathy developed, including *cis-*ptau accumulations at 6 months post injury in association with tau oligomers, tangles, and TDP-43 immunoreactive NCIs (139). As described earlier, the *cis* conformation of pThr^231^tau prevents pThr^231^ dephosphorylation and the ability of tau to stabilize microtubules. The intraperitoneal administration of a monoclonal neutralizing antibody to *cis*-ptau eliminated the pathological tau accumulation and subsequent TDP-43 pathology, suggesting a linkage between the two pathologies. However, the induction of TDP-43 pathology may in this case represent a physiological stress response marked by an upregulation in its expression as previously observed following axonal injury, and thus only indirectly linked to the induction of the tau pathology (15).

Of note, both tau tubulin kinase 1 (TTBK1) and tau tubulin kinase 2 (TTBK2) have been shown to extensively phosphorylate both TDP-43 (152) and tau (153, 154). Both kinases have been observed to co-localize with TDP-43 and tau aggregates in FTLD-tau and FTLD-TDP cases (152, 155). These observations provide further support for the concept of a pathological commonality underlying these two proteinopathies and a mechanism to drive both pathological tau and TDP-43 phosphorylation concomitantly (156).

## Conclusions

The conventional concept of ALS as a pure motor neuron disorder and a discrete disease has long been replaced by one of a multisystem disorder that is syndromic in nature and for which progressive neuropsychological deficits, including a frontotemporal spectrum disorder, are common. As this conceptualization of ALS has evolved, so too has our understanding of its underlying pathobiology as reflected in the broad range of not only causative gene mutations associated with both familial and sporadic variants of the disorder, but the broad range of protein aggregates which typify its pathology (4). Embedded within this has been the recognition that FTD and ALS are increasingly recognized to share not only a clinical phenotypic spectrum, but also an underlying pathobiology as reflected not only in alterations in the metabolism of TDP-43, but increasingly in the metabolism of tau.

While the classical role of tau is the stabilization of microtubules through interactions mediated by its MBTR, there is increasing evidence to suggest a much broader repertoire of function of tau under both normal physiological states as well as in response to stress. These functions are critically dependant on a complex array of determinants including the somatopically-specific subcellular distribution of individual tau isoforms that includes an emerging role in gene expression and inter-relationships with RNA binding proteins, the impact of cellular stress upon tau expression, the conformational structure of tau and the extent of its post-translational modification (44, 49, 157). Although a broad array of post-translational modifications and their relationship to specific disease states have been well-characterized [reviewed in (44)], we have focused on the specific role of pathological phosphorylation at Thr^175^ and the mechanisms by which this culminates in the generation of oligomeric tau and a subsequent tauopathy, including a synergistic role with pathological TDP-43 deposition.

However, several pivotal questions remain in order to further clarify the role of alterations in tau metabolism in ALS, including:

- In the context of understanding that ALS-FTSD represents a broad array of neuropsychological, behavioral, speech, and language deficits, and that our initial studies characterizing pThr^175^-tau expression were undertaken in ALS patients with cognitive impairment (ALSci) while those of Behrouzi et al. were undertaken of individuals with FTD and ALS (114), clarifying the spectrum of clinicopathological correlates to ALS-FTSD and whether alterations in tau metabolism are specific to one or more variants within the spectrum;
- Linked to this, further defining the nature of TDP-43 deposition in ALS-FTSD in tau NCI-bearing neurons with a view to understanding whether the TDP-43 accumulation bears hallmarks of pathological tau deposition or that observed in a stress response;
- Identifying the kinase or kinases responsible for the induction of phosphorylation at Thr^175^, the conditions under which the pathway becomes activated, and whether these differ amongst the range of FTLDs in which pThr^175^tau has been observed;
- Determining whether the pThr^231^tau exists in either *cis* or *trans* conformation and the associated activity of PIN1 in ALS-FTSD and its *in vivo* model as demonstrated using somatic gene transfer with rAAV9, including in the presence or absence of pathological ALS-associated TDP-43 mutations;
- Further understanding the inter-relationships between alterations in the expression of specific tau isoforms and alterations in TDP-43 metabolism, in particular in relationship to the role of TDP-43 in mediating gene expression through its nuclear functions as an RNA binding protein; and
- Determining whether therapeutic strategies targeted toward restoring physiological tau/microtubule interactions would be of therapeutic benefit in ALS-FTSD.

## Author Contributions

MS: conceptualization and original draft preparation. MS, ND, and KV: writing, review, and editing. All authors have read and agreed to the published version of the manuscript.

## Conflict of Interest

The authors declare that the research was conducted in the absence of any commercial or financial relationships that could be construed as a potential conflict of interest.
